# Airborne biological hazards and urban transport infrastructure: current challenges and future directions

**DOI:** 10.1007/s11356-016-7064-8

**Published:** 2016-06-18

**Authors:** Zaheer Ahmad Nasir, Luiza Cintra Campos, Nicola Christie, Ian Colbeck

**Affiliations:** School of Energy, Environment and Agrifood, Cranfield University, Cranfield, Bedfordshire MK43 0AL UK; Department of Civil, Environmental and Geomatic Engineering, University College London, London, WC1E 6BT UK; School of Biological Sciences, University of Essex, Colchester, CO4 3SQ UK

**Keywords:** Biological hazards, Transport, Vulnerability, Protection measures

## Abstract

Exposure to airborne biological hazards in an ever expanding urban transport infrastructure and highly diverse mobile population is of growing concern, in terms of both public health and biosecurity. The existing policies and practices on design, construction and operation of these infrastructures may have severe implications for airborne disease transmission, particularly, in the event of a pandemic or intentional release of biological of agents. This paper reviews existing knowledge on airborne disease transmission in different modes of transport, highlights the factors enhancing the vulnerability of transport infrastructures to airborne disease transmission, discusses the potential protection measures and identifies the research gaps in order to build a bioresilient transport infrastructure. The unification of security and public health research, inclusion of public health security concepts at the design and planning phase, and a holistic system approach involving all the stakeholders over the life cycle of transport infrastructure hold the key to mitigate the challenges posed by biological hazards in the twenty-first century transport infrastructure.

## Introduction

In the twenty-first century, the nature and extent of human interaction in different built environments can have a profound impact on public health. Today, we spend nearly 90 % of our time indoors in a variety of enclosed microenvironments (Buonanno et al. 2014). In broader terms, the built environment refers to any physical alteration of the natural environment through human-made structures to shelter, perform and protect their activities, ranging from dwelling and work places to recreational facilities and their supporting infrastructure. The development and expansion of transport infrastructures, especially in mega cities, has led to increased mobility of people. Today, a vast proportion of the working population spend a significant time commuting in public transport and exposure to airborne pathogens in these in-transit microenvironments is of major concern. For example, in the UK, use of public transport has increased from 9 to 11 % since 1995/97 to 2012 (DfT, 2013). However, the principles and practices in design, construction, operation and management of different built environments vary across the globe depending on a variety of factors (economic, social, political, technological and climatic) (Nasir, 2014), resulting in a range of exposure pathways and scenarios around biological hazards in these environments.

Transport built environments are subject to an array of indoor air contaminants derived from outdoor sources, building materials, furnishings, consumer products and occupant activities. These include a cocktail of particles, gases, vapours, biological agents and their derivatives. Of these, biological agents are of the greatest concern due to their allergenic, toxic and infectious potential. Enclosed environments can provide ecological niches and transmission pathways for a wide range of pathogens. Indeed, indoor environments are complex ecosystems in which there is a complicated relationship between humans, microorganisms, physical environment and structures (Nazaroff, 2016; Kelley and Gilbert, 2013; Kembel et al. 2012, 2014). Biological agents are ubiquitous in ambient environments and can enter into transport built environments through a number of routes: heating ventilation and air conditioning (HVAC) systems, doors, windows, attachment to people and objects, water infrastructure and via infected individuals and animals.

It has been recognised that overcrowding in small enclosed spaces, inadequate ventilation, recirculation of contaminated air, increased duration of exposure and susceptibility of exposed people increase the likelihood of airborne disease transmission (Nardell, 2016; Canadian Tuberculosis Committee, 2007; Li et al. 2007; Wanyeki et al. 2006). These can be influenced, to a varying degree, by design, management and operational practices in different environments. While the design and construction of the man-made enclosed spaces may be intrinsic, their operation and management is strongly influenced by a wide range of factors.

**Fig. 1:**
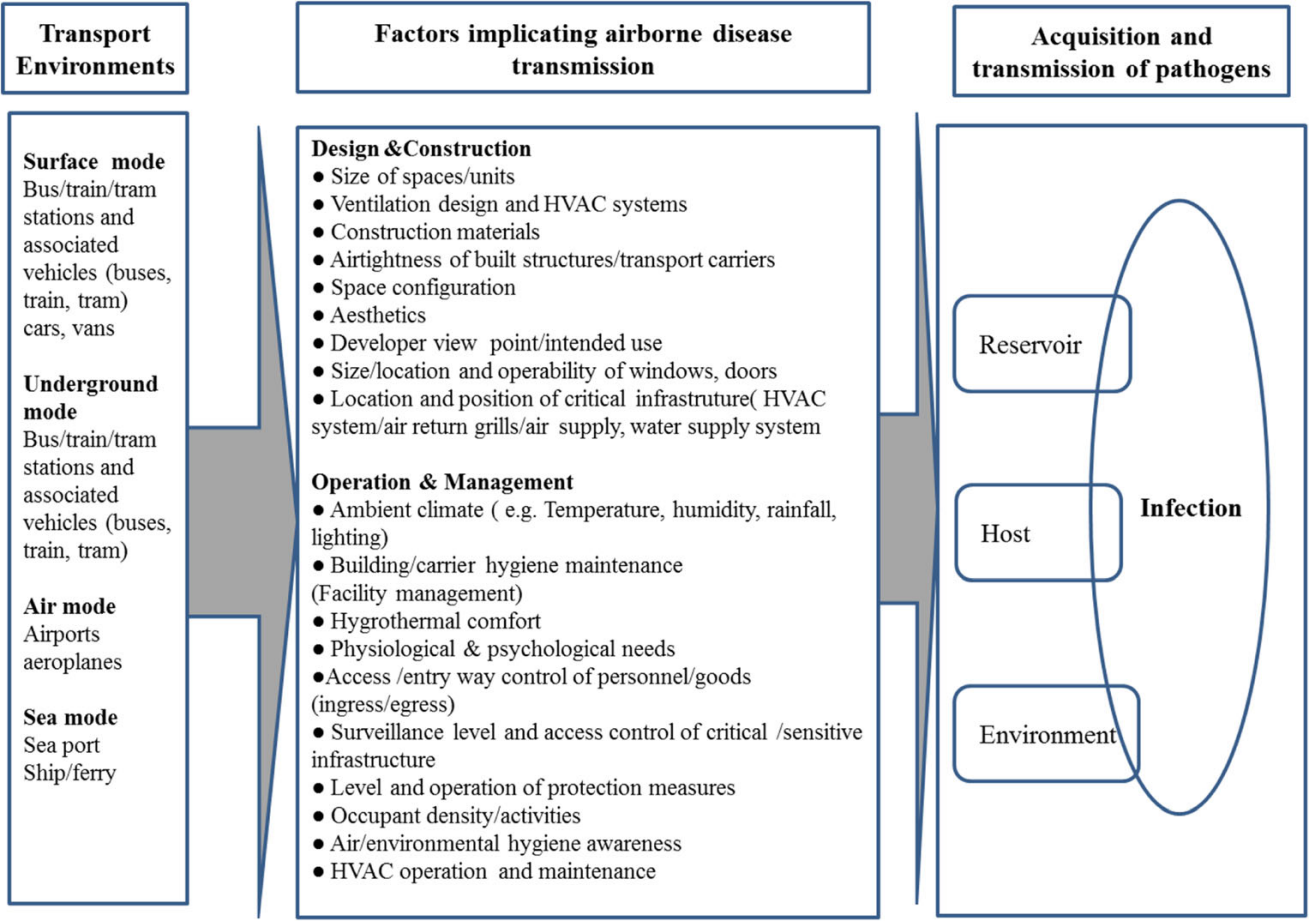
Factors influencing airborne disease transmission in transport infrastructure

Airborne disease transmission in the built environment is a complex process, and acquisition and transmission of pathogens is the end result of successful interaction between infectious agents (reservoir), hosts and transmission pathways (environment). Various elements of design, construction, use and management of different built environments can significantly impact these sub systems (sources, hosts, transmission pathways). Figure 1 illustrates how these may influence airborne disease transmission. It can be argued that the way we design, construct, operate, manage and behave inside different transport built environments can have a substantial impact on our risk of exposure to biological hazards as well as creating new exposure pathways.

In recent times, we have witnessed the emergence of new diseases (e.g. influenza H1N1 and severe acute respiratory syndrome (SARS)) and their potential to cause huge damage to societies across the globe (Holmes and Rambaut, 2004; Neumann et al. 2009). Additionally, re-emerging airborne infectious diseases, for instance, tuberculosis (TB), have a worldwide public health impact. In 2013, there were 9 million incident cases worldwide and multidrug-resistant TB (MDR-TB), extensively drug-resistant TB (XDR-TB) and TB/HIV co-epidemics are serious global health concerns (WHO, 2014). It is of note that the majority of TB incidents were in Southeast Asia, Africa and Western Pacific regions. The public transport built environments in such countries with a high burden of TB together with poor airborne disease control measures may become hubs for the airborne spread of disease. The intentional release of biological agents is also of growing concern (Cogliati et al. 2016). The 2001 bioterror incidents using spores of *Bacillus anthracis* in the USA (Canter et al. 2005) and failed bioattacks by Aum Shinrikyo in Japan (Danzig et al. 2012) offer evidence of this. Aum Shinrikyo had developed a biological and chemical weapon programme and made several failed bioattacks by weaponizing *Clostridium botulinum* and *B. anthracis*. However, in 1995, the same group released sarin gas in the Tokyo subway system, resulting in 12 deaths and over 5000 injuries (Okumura et al. 1996). Anthrax, though cannot be transmitted between humans, however has the potential to be used as a bioweapon. As early as 1953, the Ministry of Defence in the UK held tests to determine the vulnerability of the public transport to biological attack. Initially, the effects of motion and ventilation on bioaerosol dispersion were investigated, and in 1963, bacteria were released in the London Underground. This latter trial indicated that the spores can be carried for several miles on the tube system (DERA, 1999).

At present, we are living with a constant risk of an influenza pandemic, and this could have a significant effect on global public health (WHO, 2013). Additionally, transport infrastructure (railway stations, bus stations, underground trains, airports, etc.) has a high relative risk to a bioterrorist attack due to its profile, occupancy and vulnerability. There is a greater risk of exposure to airborne pathogens and likely acquisition of infection and downstream transmission of disease to the wider public due to the special characteristics of various transport means and their hubs (e.g. high population density, close interaction and complicated people movements, interconnectedness of transport network). There have been growing efforts to numerically model the risk of airborne disease transmission and propagation in both global and local transport networks (Lawyer, 2016; Zhang et al. 2016; Andrews et al. 2013; Perez and Dragicevic, 2009; Yang et al. 2008). It is fair to argue that the uncertainty about when a pandemic or bioterror attack may occur and the unpredictability about the severity of such an incident leave no option but to prepare in advance. Therefore, it is time to rethink the role of transport built environments in airborne disease transmission. Specifically, we need to ask the following: What are the existing practices in design, construction, use and management in these environments and their implications in airborne disease transmission? Are we inadvertently creating a transport infrastructure which facilitates exposure to airborne biological hazards? What may be needed to prevent a drug-resistant disease to spread from person to person versus preventing a terrorist attack? What should be the main focus in design and management? What are the potential research areas to focus on in order to enhance the resilience of transport infrastructure to biological hazards?

This paper aims to review the existing evidence on disease transmission in transport built environments with a view to highlight the factors increasing the vulnerability of them to disease transmission, discuss the potential protection measures and identify future direction for research to enhance the resilience of transport infrastructures to biological hazards. This will not only add to the knowledge on the current vulnerability of both occupants and transport infrastructure to airborne disease transmission, but also assist in identifying the gaps where interventions can be made to build a bioresilient transport infrastructure. In addition, it can inform the future research agenda to allow us to anticipate, prepare for, and control future epidemic outbreaks or intentional release of biological agents in critical infrastructure.

## State of the art

Overall, the scientific literature on airborne infection transmission in various transport hubs and transport means is limited. Reports are available on air/droplet disease transmission (TB, SARS, influenza, measles) during commercial air travel. A review on transmission of respiratory infections during air travel by Ledler and Newman (2005) provides a detailed knowledge on aircraft cabin environment/operation and reports on outbreaks of respiratory illness. They concluded that due to existing guidelines from the World Health Organisation (WHO) for prevention and control of respiratory illness during air travel and controlled cabin environments (e.g. up to 20 air changes per hour and HEPA filtration), the overall risk due to air/droplet borne disease transmission is low. However, the proximity to the source and duration of exposure increases the risk. The stage of illness and size of aircraft were also mentioned as influencing factors. SARS outbreaks during air travel highlighted the likely airborne transmission and a wider zone of high-risk environment than indicated by the WHO and other organisations (e.g. an increased risk of transmission is associated with sitting within two rows of an infected person for more than 8 h). Similar conclusions were drawn in another review of infectious disease transmission during commercial air travel by Mangili and Gendreau (2005). With reference to transmission of TB during commercial air travel, a systematic review by Abubakar (2010) highlighted the limited evidence on TB transmission.

The European Centre for Disease Prevention and Control (ECDC) has extensively reviewed the literature on infectious disease transmission in aircraft, assessed the risk associated with transmission of different infectious agents onboard aircraft and formulated guidelines on their control (Leitmeyer, 2011). These have highlighted that although available literature suggests the frequent transmission of TB, influenza, SARS, meningococcal disease and measles, there is scarcity of data on confirmed on board transmission of infectious diseases. Infectivity of index case, susceptibility of contacts and effectiveness of exposure in terms of proximity, duration and cabin air quality were identified as factors influencing onboard transmission of infectious diseases. Recently, an Australian study to quantify the risk of measles transmission on aeroplanes during 2007–2011 found that risk was not associated with seating proximity (Hoad et al. 2013). Likewise, Young et al. (2014) concluded proximity had no impact on the risk of infection by influenza. A report on influenza transmission on aircraft by Moser et al. (1979) illustrated the significance of ventilation. In this case, an aircraft was delayed for 3 h in an Alaskan airport and remained on the ground with the ventilation system turned off. Seventy-two of the 54 passengers developed influenza due to one index patient on board. Consequently, it is now recommended that adequate ventilation must be supplied in ground delays of more than 30 min.

A number of studies on respiratory infection (influenza, TB, Legionella) in ship travel (passenger, cargo, naval) have been reported (Beyrer et al. 2007; Brotherton et al. 2003; CDC 2010; Jernigan et al. 1996; Joseph et al. 2007; Kak, 2007; Kura et al. 2006; Lim, 2011; Schlaich et al. 2009; Tarabbo et al., 2011; Vera et al. 2014; Ward et al., 2010). Isolated environments with close interaction among a large number of individuals and shared facilities (water and air conditioning) have been identified as factors that increase the risk of exposure to airborne infections in these settings. A typical example of airborne infection is an outbreak of Legionnaires’ disease. Here, the most common environmental factors that elevate the risk of exposure are contaminated water supply systems, spas and pools and air handling systems (Beyrer et al. 2007; Jernigan et al. 1996; Kak, 2007; Kura et al. 2006). Spread of TB is another example of airborne infection transmission. A study by Houk (1980) on TB spread in a naval ship concluded that infection transmission was due to rapidly and evenly dispersed infectious droplet nuclei throughout a closed environment with a recirculation ventilation system. Lim (2011) has noted proximity and social interaction among a large number of people, long duration of cruises, mixing of passengers from Southern and Northern hemispheres and arrival of new susceptible passengers on subsequent journey legs as specific vulnerabilities of cruise ships to influenza.

With reference to ground transport, a review by Mohr et al. 2012 has reported 14 events of airborne infection transmission in public transport (commuter buses, school buses, train). Of these, eleven were of TB, two were meningococcal and one involved measles and most of these were in school buses. Additionally, the authors have also presented non-scientific literature (Google News, Google Scholar, GENIOS and World News) on airborne infection in public transport (eight events on TB, SARS, meningococcal disease and Rubella). Poor ventilation (windows and doors closed due to outside weather), ventilation systems (recirculation), proximity to index cases (crowding) were found as environmental factors influencing the risk of airborne infection transmission on public ground transport. Similarly, a systematic review on TB transmission on public transport (school buses, train, commuter van) has reported that contact investigations on these conveyances found a positive tuberculin skin test (TST) in 10–78 % of the persons travelling with the index case (Edelson and Phypers, 2011). They also highlighted that poor ventilation, closed ventilation systems and proximity to index cases increases the risk of exposure to TB. Studies from countries with high TB incidence has shown that public transportation (often crowded and poorly ventilated) may play a critical role in transmission and sustaining TB infection (Andrews et al. 2013; Horna-Campos et al. 2010, 2007). A case control study from Nottingham, UK, has shown that recent use of public buses and trams is a significant individual risk factor for the acquisition of acute respiratory infection (leading to GP consultation) in winter (Troko et al., 2011). Reports have implicated the role of train travel during the 2009 influenza A (H1N1) pandemic (Cui et al. 2011; Pestre et al. 2012). Numerical modelling studies have also been carried out to quantify the risk of airborne infection (Furuya, 2007), dispersion of infectious droplets in trains (Zhang and Li, 2012) and propagation of airborne disease via public transport at city level (Zhang et al. 2016). No publications were found on airborne infection transmission on the underground or in any of the transport hubs such as stations or airports. In a review on health and safety hazards associated with subways, Gershon et al. (2005) remarked that although infectious disease transmission in subways is conceivable, this has not been documented. While more recently, Zhao et al. (2015) have utilised urban subway mobility data to model the risk of an epidemic transmission via the Beijing subway.

## Vulnerability of transport infrastructure to airborne disease transmission

**Fig. 2:**
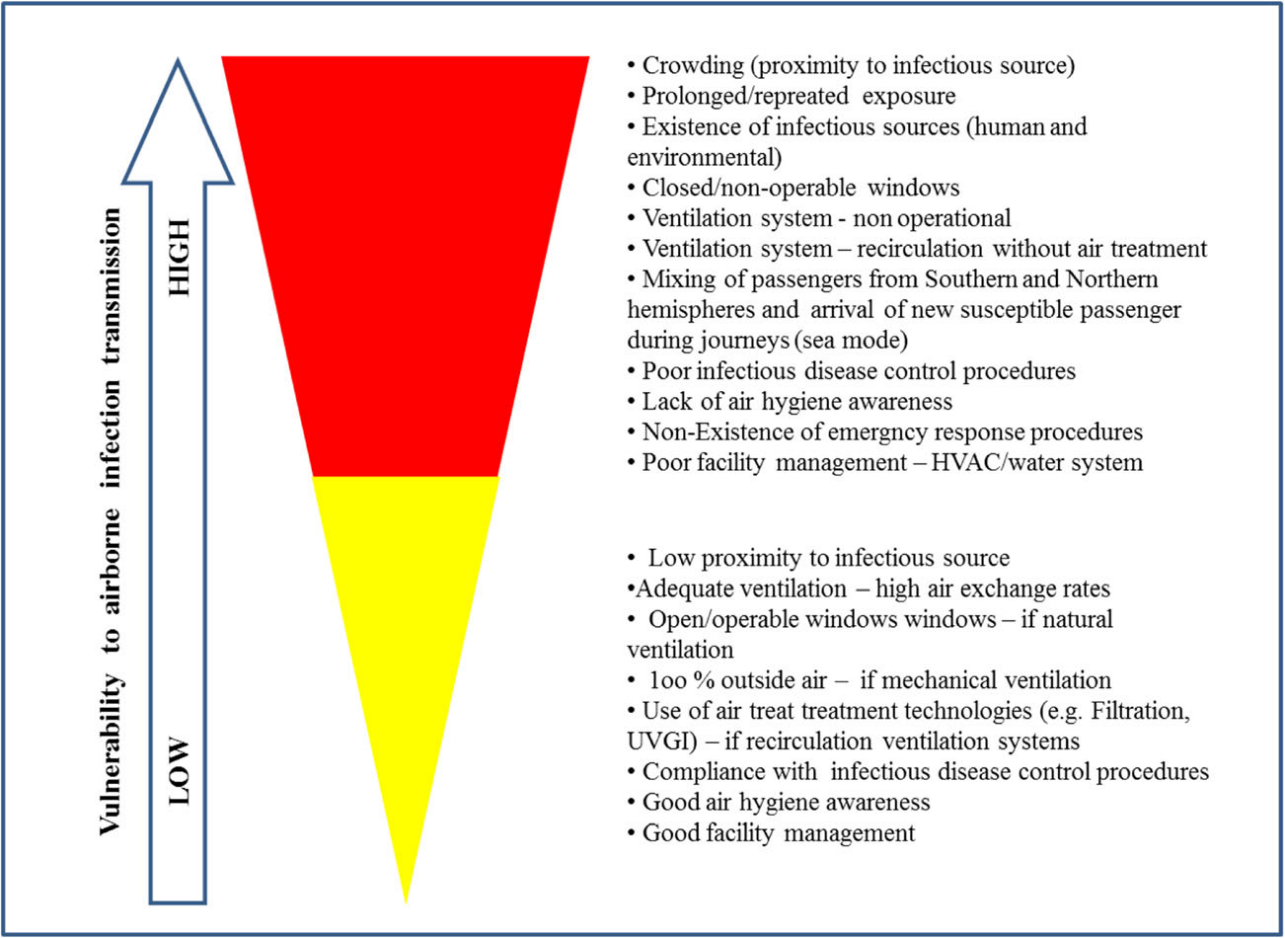
Vulnerability profile of transport infrastructure to airborne disease transmission

The available literature on airborne infectious disease transmission in transport built environments, though scanty, offers valuable knowledge on factors enhancing risk of exposure to biological hazards. Although the existing literature is not sufficient to quantify the association of different environmental factors to disease transmission in transport built environments, it can be used to develop a qualitative vulnerability profile of transport built environments to airborne disease transmission (Fig. 2).

In transport built environments, humans and environmental sources (mobile and fixed) are the major reservoir of biological agents. Respiratory droplets produced by infected individuals during different expiratory activities (talking, singing, coughing, and sneezing) may contain pathogens. These droplets either settle or remain suspended in the air as droplet nuclei depending on their composition and size at the time of release and hygrothermal condition of the built environment. These are subject to same aerodynamic processes that govern abiotic particles in an enclosed space (Nazaroff, 2016). Small droplet nuclei (<5 μm) can remain suspended in the air for a long duration due to their low settling rates and can be transported to other areas away from the source (e.g. by air currents or recirculation ventilation). The settled droplets (including those larger than 5 μm) contaminate the surfaces and may transfer to susceptible hosts by surface-borne pathways or resuspend again after evaporation. Type and frequency of respiratory activities, site of infection, pathogen load and type are critical factors that affect the probability of infection transmission by the airborne route (Gralton et al. 2011). The potential environmental sources include but are not limited to HVAC systems, water reservoirs and distribution systems, maintenance activities and contaminated surfaces. In addition, deliberate release of infectious agents in any transport mode or hub have great potential to start the chain of airborne infection transmission.

It is worth highlighting that in addition to the airborne route, the dispersion and transfer of infectious agents deposited on various surfaces/materials/matrix (e.g. skin or in respiratory secretions, to hands and/or to high-touch surfaces—doorknobs, staircase railings, seats, escalator hand rails, chair arms, grab rails, cash machines, phone, ticket machines) also offer a major transmission pathway. For example, studies have shown the contamination of public buses in two major cities in Portugal with MRSA (Conceição et al. 2013). In fact, it can be argued that successful airborne infection transmission from source to susceptible host is a complex and multifaceted process which can involve both airborne and surface borne pathways and it is difficult to disentangle the two.

Proximity to infectious source and duration of exposure are the major variables in risk of airborne disease transmission. In various modes of transport and their hubs, the duration of exposure to infections sources can be highly variable. It may involve a single trip (long or short) or multiple trips (repeated/cumulative exposure). The WHO recommends the contact tracing of the individuals who were in close proximity (within two rows) of an infectious TB person for more than 8 h during air travel (WHO 2008). However, transmission during short but intensive and repeated exposure (e.g. school and mini buses) in ground transport has been reported (Golub et al. 2001; Horna-Campos et al. 2007; Mohr et al. 2012). This clearly highlights that successful airborne infection transmission can occur during short exposure periods depending on the characteristics of infectious source (pathogen load), host (susceptibility) and environment (proximity, pathogen concentrations). However, the relationship among these variables remains poorly understood.

Crowding is a common feature in various transport modes and transport hubs. For example, at present, London is facing chronic overcrowding on public transport, especially during peak rush hours. The results of a survey (Spring 2011) by the Department for Transport (DfT) revealed that the top ten overcrowded services were between 47 and 66 % over their capacity limit (DfT, 2011). Not only are the train carriages overcrowded but also the stations. During the rush hours, commuters are sitting and/or standing in close proximity under poorly ventilated conditions and this may have serious implications for airborne disease transmission. For instance, travelling with symptomatic individuals, especially during pandemics, in crowded and poorly ventilated public transport could increase the risk of infection transmission via direct and indirect contact.

## Protection measures

Despite the limited knowledge on quantitative association among different elements of design, construction, use and management with airborne disease transmission in transport built environments, the available evidence emphasises their vulnerability to different airborne biological hazards. A range of protection measures are available to improve the resilience of transport built environments to airborne biological hazards.

**Fig. 3:**
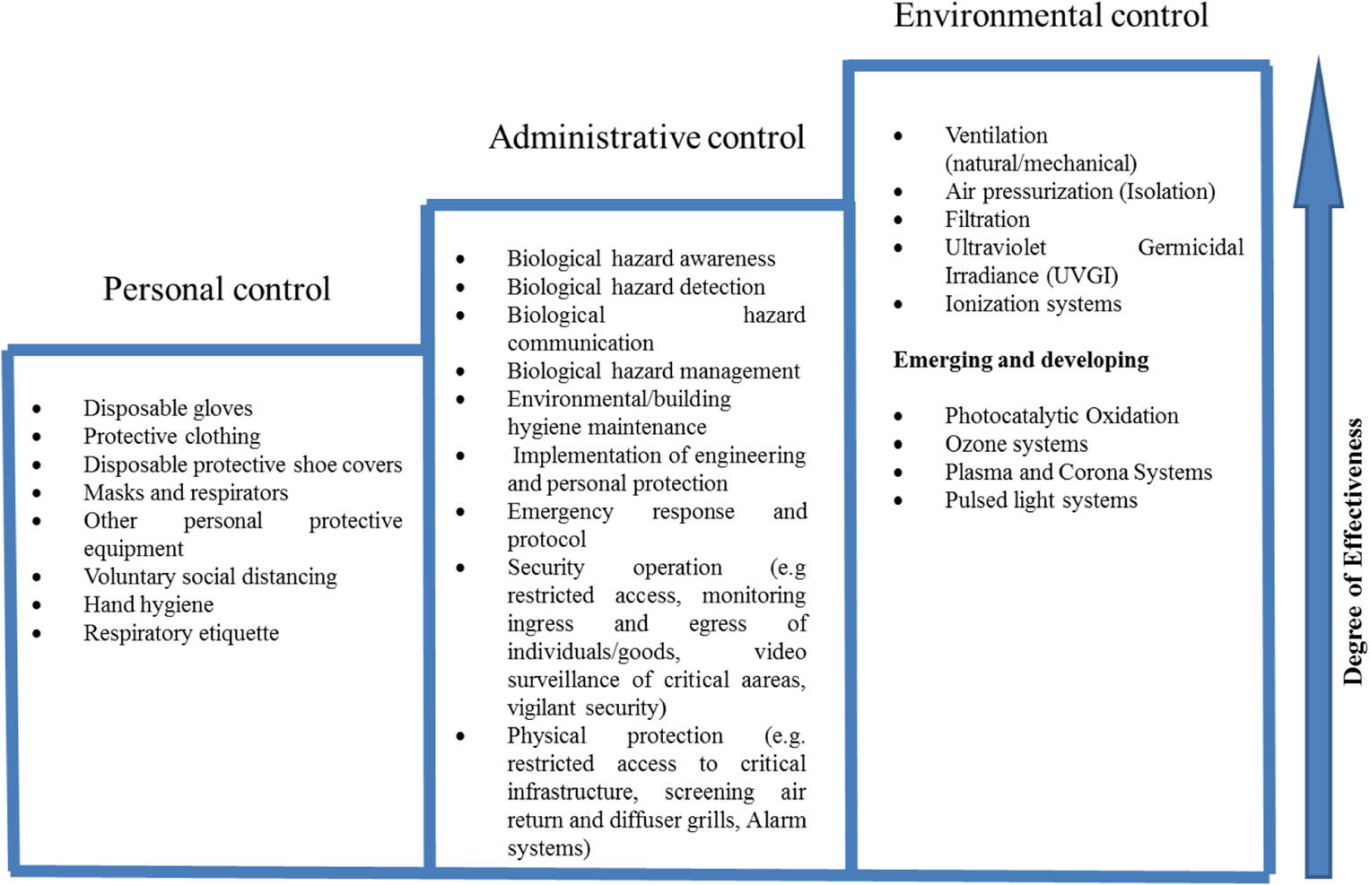
Hierarchy of control measures to enhance resilience of transport infrastructure to airborne biological hazards

The approach to minimise the risk of acquisition of airborne infection entails deployment of control measures that break the chain of transmission. A good body of knowledge is available on airborne infection control technologies and strategies for enclosed spaces, particularly, in health care built environments (Kowalski, 2012; Azimi and Stephens, 2013). These can be adapted to transport built environments. These include administrative, environmental/engineering and personal protection. Administrative controls are comprised of policies and procedures and their implementation to reduce opportunities for infection occurrences and cross infection. Environmental/engineering controls focus on reducing the concentration of infectious agents and are either integrated into HVAC systems or installed/fixed in indoor spaces. Additionally, there has been a growing literature on use and efficacy of these technologies in portable devices (Verhougstraete and Reynolds, 2016; Boyce, 2016; Gunschera et al. 2015; Zuraimi et al. 2011; Zhang et al. 2011; Chen et al. 2010; Grinshpun et al., 2007). Personal protection controls are used in high-risk environments and emergency scenarios where administrative and environmental controls cannot adequately offer protection. Figure 3 depicts the hierarchy of control measure to enhance resilience of transport infrastructure to airborne biological threats.

Due to growing international travel and the emergence of diseases with potential to be global public health threats, the WHO has revised International Health Regulations (IHR) which were adopted in May 2005 and entered into force on June 2007. These offer guidelines towards prevention, protection, control and public health response to global spread of disease without compromising international trade and traffic (WHO 2005). A recent report by the Airport Cooperative Research Programme (ACRP) evaluated the risk of infectious disease transmission via droplet, airborne and contact modes within airports and aboard aircrafts and identified 24 mitigation measures classified into three broad categories: buildings, airplanes and people (TRB, 2013). This report recommended the use of hand sanitizer stations at strategic locations inside buildings, use of broad spectrum disinfectants, availability of biohazard kits, hand-free bathroom appliances, hand-free transaction tools and appropriate operation and maintenance of HVAC systems (ventilation, filtration). The use of upper room ultraviolet light (UVC), especially for high-risk (quarantine, isolation) and high-density areas (queuing areas), was also suggested. For aeroplanes, the mitigation measures included decreasing ventilation downtime (e.g. parked at gate), availability of biohazard kits, use of hand sanitizer during and after the flight and use of broad spectrum disinfectants. With reference to people, the highly recommended actions were implementing campaigns on becoming a healthy traveller and healthy worker and on seasonal influenza vaccination and hand hygiene/cough/sneeze etiquette.

In order to prevent inanimate surfaces acting as reservoirs of pathogenic organisms, the use of antimicrobial coatings on different high-touch surfaces is also gaining attention. A range of coatings/polymers have been tested on different surfaces—plastic, glass, steel, natural leather (Wei et al. 2014; Pollini et al. 2013), and a growing body of evidence is available on the efficacy of antimicrobial coatings, particularly, in health care built environment (Boyce, 2016; Casey et al. 2010; Page et al. 2009). At present, a range of antimicrobial coatings has been marketed and can be adopted appropriately to different transport microenvironments. The use of antimicrobial copper has been reported at border control counters at the Arturo Merino Benítez airport and metro train network in Chile (Copper Development Association, 2013, 2014). Surfaces in the Hong Kong metro have been coated with nano-based disinfectants (Davies, 2007).

Taylor et al. (2013) reviewed different risk assessment methodologies, guidelines, recommendations and tools/software to assess, prevent and mitigate the potential impact of building vulnerability to bioterror attacks. They introduce a framework to classify different protection measures and highlight the inter relationship between different protection measures and their impact on overall building vulnerability and resilience to bioterror attack. Recently, the Federal Highway Administration, U.S. Department of Transportation, has developed a learning tool to inform transportation agencies about plans and responses in biohazard events. The tool contains detailed information about potential biohazard agents, events, release scenarios in different modes of transport, existing guidelines and analytical tools framing emergency response to biohazard events in different transport modes and identified various information system technologies to enhance preparedness and response actions (FHWA, 2015). Preventing, mitigating, monitoring and responding to biological threats in transport infrastructure is a complex task and requires a multidisciplinary alliance to design and implement appropriate protection measures which must be informed by venue and scenario rather than supply driven. Facility management departments/divisions have a major role in ensuring the operation and efficacy of various control measures. Particular attention is required to keep in view the interactions and interdependencies between different components of transport infrastructure in order to overcome any unintended consequences of a control measure.

## Potential research areas

In the twenty-first century, we are facing threats from a range of airborne biological hazards (e.g. pandemics, natural disasters, bioweapons). The social and economic impact of incidents of influenza pandemic, SARS outbreak and anthrax attacks in the recent past clearly highlight the scale of threat to humanity from biohazards. The critical role of transport infrastructures in the healthy functioning of an urban environment puts it at a higher risk to biohazards and warrants a need to develop and adopt such tool/strategies/protection measure that inform and assist in building bioresilience to transport infrastructure. In recent times, a rapid change in policies, laws, and standards has been seen due to growing international concerns about the impact of built environments on natural resource depletion and degradation, waste generation and accumulation, ecosystems and global warming. Consequently, energy efficiency and sustainability has emerged as a guiding paradigm for twenty-first century built environments. The growing emphasis on energy efficiency and the resultant changes in design, construction and operation of various transport built environments (e.g. airtight structure, high space usage efficiency) may lead to increased vulnerability of these built environments to airborne disease transmission. We believe to move towards ‘healthy transport infrastructure’ health and well-being of occupants and environmental hygiene should be the primary objective of all the policies and regulations, and this entails a collaboration among policy makers, architecture/engineering/construction industry, manufacturers and vendors supplying the building technologies, systems, products and materials and public health officials.

Over the last decade, a growing body of knowledge has been created on airborne biological contaminants in built environments primarily on two independent streamlines by biosecurity experts and public health practitioners. But is it possible to build bioresilient cities by taking such an approach? In our view, the challenges posed by biological hazards in today’s world are multifaceted, and surely, such a divided approach is bound to fail. This situation calls for unification of security and public health research scholarship—*public health security*. What is needed is a holistic system approach involving close interaction among all the stakeholders (architects, urban planners, public health practitioners, biosecurity experts, law experts). It is vital to incorporate the concept of public health security into the life cycle phases of critical urban infrastructures, including transport, from design and planning to upgrading/decommissioning.

Despite the growing knowledge on dynamics of airborne disease transmission in transport infrastructures, there are certain areas which need focus in order to develop a public health security index for critical urban infrastructure and an integrated tool box (with tools for exclusion, detection, mitigation, response, decontamination) for public health security. Table 1 shows the areas to be developed under a future research agenda in order to improve public health security.

**Table 1:** Highlights of future research areas and research focus

Research area	Research focus
Transmission of airborne disease or release of biological agents of concern	What are the mechanisms of airborne disease transmission in transport infrastructures? What is the role of different environmental factors in transmission? To what extent do various design and operation practices provide or limit exposure pathways to airborne biological hazards?
Human environment interaction	What are the flow patterns of people and their interactions within transport infrastructures (how many, where, how long)? What are the characteristics of high-risk space-people interaction? How can we gauge the relationship of complexity and interconnectivity of urban transport infrastructures and the exposure pathways to biological hazards?
Hazard analysis and critical control points	Which methods/tools are required to predict the spread of biological agents and identify critical control points and their spatio-temporal dispersion in urban transport infrastructures?
Public health security and ethics	How can the biosecurity agenda serve the public health agenda? What ethical issues need to be considered in surveillance for biological threats?
Detection and diagnostics of biological hazards	Development of rapid detection of airborne biological hazards Development of attributed systems of biological detection Instrumentation to facilitate measurement of individual exposure during use of transport infrastructures
Mathematical modelling	Airborne dispersion of biological agents in transport specific infrastructures and resultant public health impact. Modelling of passenger movement and proximity in transport infrastructures.
Prediction and tracking	How various existing technologies (e.g. remote sensing, geospatial technologies) can be used as a tool to predict vulnerabilities and track the intermodal dispersion of biological agents?
Design and engineering	What are the functional and non-functional requirements for different engineering controls and how can design and engineering science inform the optimal balance between air hygiene and energy efficiency? Operational effectiveness of protection measure vs cost effectiveness.

## Conclusions

The design, construction, use and management of transport infrastructures, in particular, public transportation systems/hubs, can greatly impact airborne disease transmission. The environmental conditions and human interaction within different means of transport and their associated built environments can lead to successful transmission of airborne diseases. The advancement in means of international travel, in particular, air travel, may facilitate and influence the propagation of already known pathogens and emergence of new pathogens. International transport hubs hold the potential to act as hubs for the global spread of airborne diseases such as SARS and H1N1 influenza. Additionally, their critical role and operation in urban environments also make them a high-risk infrastructure to bioterror attack.

In general, the literature on airborne infection transmission in various transport infrastructures is limited and mainly focused on commercial air travel. However, the available knowledge offers valuable insights on potential factors enhancing risk of exposure to biological hazards in transport infrastructure. This can be translated to develop a qualitative vulnerability profile of different transport built environments and to design intervention strategies to enhance resilience towards airborne biological hazards. Breaking the chain of infection transmission is critical to the success of any airborne infection control intervention and knowledge and practices from health care built environments to control airborne infection (administrative, environmental/engineering and personal protection) can be of great relevance to transport built environments.

In the wake of new and emerging diseases, the rising number of drug-resistant pathogens and an ever looming threat of bioweapons the vulnerability of transport infrastructures to airborne disease transmission can have severe implications. Hence, it is time to redefine the concept of national security and public health by bringing these two in close interaction in order to build bioresilient cities. A holistic system approach, which takes into account all the factors influencing airborne disease transmission in transport infrastructures, can surely advance our capabilities to enhance prophylactic public health security management. By designing and implementation of sustainable interventions, we can not only reduce the current vulnerability of transport infrastructures to airborne disease transmission, but also better prepare for future biological threats, both epidemic outbreaks and bioterror attacks.
